# The Hospital World

**Published:** 1910-10-29

**Authors:** 


### THE HOSPITAL WORLD.

### THE LATE PRINCE FRANCIS OF TECK.

It is no conventional tribute to say that the hospital world is the chief mourner in the nation, and the chief sympathiser with the Queen, in the death of her brother, Prince Francis. When he became a governor of the Middlesex Hospital nine years ago it could hardly have been expected that he would perform so great a work as he actually did for the institution. But the unobtrusiveness of his entry into hospital work was the first step in a deliberate policy by which the Prince mastered the duties, in turn, of hospital governor, vice-president, and deputy chairman of the board of management. This laid the foundation of his practical experience, and his election last March to the office of chairman, in succession to Lord Cheylesmore, gave to this experience the opportunity to which his high rank entitled him. The union in an administrator of a personal knowledge of routine with an hereditary position of the highest influence proved invaluable. The amount of work got through, and the success which attended it, have been alike astonishing. It will come almost as a surprise to our readers to be reminded that it is only six months since Prince Francis became chairman, so crowded with important business have they been. The launching of the appeal for £20,000 which confronted him on his election was no easy matter, at a time when public opinion was distracted with other matters, at a time too when the national mourning was, so unexpectedly, at hand. Nevertheless, in three months all difficulties as regards the raising of the money were overcome. The second stage of the appeal, the increasing of the number of annual subscribers to preserve the solvency attained by stage one, then faced him. This represented the collection of sufficient promises of yearly payments—let these words be weighed carefully—to guarantee an increased income of £7,000 or £10,000 a year. Hospital secretaries will want no reminder that it is easier to gain ten donations than five annual subscribers. His efforts to attract them, however, were interrupted by his death. The vast problems opened up by Mr. H. I. Barnato's bequest, the questions centring round the cancer charity and its relation to the Middlesex Hospital, at the same time engaged him. The result was that he himself laid the foundation stone of the Barnato-Joel Memorial on July 14 last. This was a solution in which business ability had a large share, for bequests so enormous as Mr. Barnato's are not trusts to be accepted lightly. The Convalescent Home at Clacton-on-Sea, the sports ground at Park Royal for the medical students, owe much to his interest—indeed the latter hardly could have been purchased without him. This is a record, not sought out but publicly accessible, of Prince Francis of Teck's chairmanship of six months' duration. Beside it, comment is superfluous. This is the only aspect of his life with which we are here concerned, and in the hospital world certainly he will be honoured by his fellow-workers—in a real sense that is, judged by his peers.

### THE FUNERAL.

The burial of Prince Francis of Teck took place on Wednesday last at Windsor. The coffin was removed early in the morning from Marlborough House Chapel to Paddington Station, attended by Prince Alexander of Teck, Lord Colebrooke (Lord-in-Waiting), the Hon. Sidney Greville (Groom-in-Waiting), representing the King, and Colonel Frank Dugdale, representing the Queen, together with Sir Douglas Dawson and Captain Fitzwilliam.

At 10.45 the special train, conveying the coffin, left Paddington Station for Windsor. The King and Queen, the Duke and Duchess of Teck, and other members of the Royal Family were on board the special train, which was preceded by another special carrying those invited to attend the ceremony in St. George's Chapel. Among these latter were members of the administrative and medical staffs of the Middlesex Hospital, the Duke of Northumberland, K.G., President of the hospital; Lord Grenfell of Kilvey and the Hon. G. W. Spencer Lyttelton, representing the Vice-Presidents of the hospital; Messrs. G. J. Majoribanks and C. H. Burnand, treasurers; Colonel Gathorne-Hardy, Deputy-Chairman; Sir Squire Bancroft, Lt.-Colonel Lambert Mears; Messrs. Gillett, Houghton, Hudson, Cavendish-Bentinck, Debenham, Ormerod, Hoare, G. Cunningham, Charles Davis, and Colonel Charles Needham, representing the Weekly Board of Governors; and Mr. Clare Melhado, the Secretary-Superintendent; Drs. Fowler, Pasteur, and Thomson, and Messrs. Pearce Gould, Bland Sutton, Somerville Hastings, and A. E. Johnson, representing the medical and surgical staff; Rev. H. E. Gunson, Chaplain to the hospital; and Miss A. Lloyd Still, Lady Superintendent of the hospital.

On its arrival at Windsor the coffin was placed on a gun-carriage, the following being the pall-bearers: Sir George Arthur, Lord Annaly, Lord Kitchener, Earl of Kilmorey, Hon. Sidney Greville, Lord Vane-Tempest, Lord Sandhurst, and the Marquis of Ripon. The King, the Duke of Teck, and Prince Alexander of Teck walked behind the coffin, followed by the Norwegian Minister, representing the King and Queen of Norway; Earl Howe, representing Queen Alexandra; the German Ambassador, representing the German Emperor; and the Austro-Hungarian Ambassador, representing the Emperor of Austria. On the coffin rested the insignia, war medals, helmet, and sword of the Prince.

The nave was lined by non-commissioned officers and men of the 1st (Royal) Dragoons and of the King's Royal Rifle Corps. Mendelssohn's Funeral March was played; and as the procession moved up the chapel the choir chanted the sentences by Croft.

Representatives of the various regiments, schools, organisations, and clubs with which his Serene Highness had been associated during his lifetime were present at the funeral service. A large number of wreaths and other floral tributes had been forwarded to Windsor, among them being tokens of regret from the members of the Weekly Board of Governors of the Middlesex Hospital, "with deep sympathy and affectionate remembrance"; "In memory of a true friend," from the students of the Middlesex Hospital; from the officers, cadet non-commissioned officers, and cadets of the Middlesex Hospital Company of the University of London Officers' Training

Corps, "as a humble tribute of respect and loyal devotion"; from the members of the medical and surgical staff of the Middlesex Hospital, "in token of their deep and lasting regard"; from the secretary-superintendent and members of the administrative staff of the Middlesex Hospital, "with respectful and heartfelt sympathy"; from the matron, sisters, and nurses of the Middlesex Hospital, "with deep and lasting sorrow"; from the patients of the Middlesex Hospital, "in remembrance of a true friend to the poor"; "with dutiful and grateful remembrance," from the matron, patients and staff of the Middlesex Hospital Convalescent Home, Clacton-on-Sea; from Mr. and Mrs. F. Clare Melhado, "with deep and heartfelt feelings of sorrow."

An Extraordinary Court of Governors was held in the Board Room of the Middlesex Hospital on Friday last, the 28th inst., at 12 noon, for the purpose of voting humble addresses of condolence on the deeply-lamented occasion of the death of Prince Francis of Teck, G.C.V.O., Vice-President and Chairman of the Weekly Board of Governors of the hospital. The Court was presided over by his Grace the Duke of Northumberland, K.G., President of the hospital.

### Personalia.

The King has become patron of the West London Hospital, Hammersmith Road, London, W.

The King and Queen have become patrons of the Victoria Hospital for Children, Chelsea, and his Majesty has become patron of the London Lock Hospital, Harrow Road, W.

Mr. Edwin Morris, J.P., chairman of the Aberystwyth Union, has been appointed its representative on the committee of the Welsh National Memorial to King Edward VII.

Prince Arthur of Connaught has accepted the office of President of St. Mary's Hospital, Paddington, and was formally elected at a quarterly Board of Governors held at the hospital last week.

The monthly meeting, held only a few days ago, of the board of management of the Royal Victoria Hospital of Belfast had to record the death of one of its oldest members. Mr. William M'Neill, of University Road, had been for a quarter of a century a member of the board, and though at a later period he had not been able to take so active a part in the routine work of the committee, there was a time, as some will remember well, when he was both interested and energetic. A resolution of sympathy with his relatives was passed.

Mr. Ralph Slazenger, of the well-known firm of tennis-ball manufacturers, and lately a sheriff of the city, has died at his house in Kensington. He was a governor of several charities, but the institution most favoured by him was the Royal Orthopædic Hospital. Would it be fanciful to suggest that orthopædic cases would excite more than any others the sympathies of a man so interested in athletics? Whatever the reason, however, the Royal Orthopædic Hospital received several benefits, and much liberality from Mr. Slazenger, whose memory it will preserve for many a day.

Mr. Alderman Thomas Crew, J.P., Mayor of Macclesfield has resigned his governorship of the Macclesfield Infirmary. This has been accepted, but with real regret. For many years Mr. Crew has been one of the most useful members of the governing body, and a few years ago, on his seventieth birthday, generously gave £500 to the endowment of a bed. Mr. Lockett, J.P., who presided at the monthly meeting at which the letter of resignation was read, and Mr. Grafton, applauded the proposal to accompany the acceptance with an appreciation of the great services Mr. Crew has performed for the hospital.

### Elections and Appointments.

The Marquis of Hertford has been re-elected President of the Birmingham Orthopædic and Spinal Hospital.

Mr. G. S. Battle has been appointed a member of the Weekly Board of the Lincoln County Hospital, in place of the late Alderman W. H. Morton.

At the monthly meeting of the Executive Committee of the Worcester General Infirmary the principal business was to confirm the appointment of Mr. Shrewsbury Smith as vice-chairman, and also to pass a vote of condolence with the relations of the late Major Hill, who was for many years a member of the committee and a staunch friend to the Infirmary.

